# RNA Interference Screening Identifies Novel Roles for RhoBTB1 and RhoBTB3 in Membrane Trafficking Events in Mammalian Cells

**DOI:** 10.3390/cells9051089

**Published:** 2020-04-28

**Authors:** Maeve Long, Tilen Kranjc, Margaritha M. Mysior, Jeremy C. Simpson

**Affiliations:** Cell Screening Laboratory, School of Biology and Environmental Science & Conway Institute for Biomolecular and Biomedical Research, University College Dublin, Belfield, Dublin 4, Ireland

**Keywords:** Rho GTPase, RhoBTB1, RhoBTB3, Golgi, membrane traffic, RNAi screening

## Abstract

In the endomembrane system of mammalian cells, membrane traffic processes require a high degree of regulation in order to ensure their specificity. The range of molecules that participate in trafficking events is truly vast, and much attention to date has been given to the Rab family of small GTPases. However, in recent years, a role in membrane traffic for members of the Rho GTPase family, in particular Cdc42, has emerged. This prompted us to develop and apply an image-based high-content screen, initially focussing on the Golgi complex, using RNA interference to systematically perturb each of the 21 Rho family members and assess their importance to the overall organisation of this organelle. Analysis of our data revealed previously unreported roles for two atypical Rho family members, RhoBTB1 and RhoBTB3, in membrane traffic events. We find that depletion of RhoBTB3 affects the morphology of the Golgi complex and causes changes in the trafficking speeds of carriers operating at the interface of the Golgi and endoplasmic reticulum. In addition, RhoBTB3 was found to be present on these carriers. Depletion of RhoBTB1 was also found to cause a disturbance to the Golgi architecture, however, this phenotype seems to be linked to endocytosis and retrograde traffic pathways. RhoBTB1 was found to be associated with early endosomal intermediates, and changes in the levels of RhoBTB1 not only caused profound changes to the organisation and distribution of endosomes and lysosomes, but also resulted in defects in the delivery of two different classes of cargo molecules to downstream compartments. Together, our data reveal new roles for these atypical Rho family members in the endomembrane system.

## 1. Introduction

Trafficking within the endomembrane system of eukaryotic cells relies on a tight cooperation between lipids, proteins, and cytoskeletal elements working together in a highly organised manner to sort and secrete newly synthesised proteins, internalise and recycle nutrients, as well as regulate membrane-bound organelles and cellular signalling pathways [1].

Within the endomembrane system, the early secretory pathway encompasses the endoplasmic reticulum (ER), the site of protein synthesis and folding, and the Golgi complex that modifies and processes the newly formed proteins received from the ER. Such ER-to-Golgi forward transport is counterbalanced by the retrograde pathway, which maintains the continuous recycling of proteins and membranes from the endosomes and Golgi to the ER, and the endocytic pathway that ensures internalised macromolecules and membrane from the plasma membrane traffic to the lysosomes by passing through the endosomal system [2,3,4,5]. These trafficking steps are widely known to be regulated by members of the Ras family, in particular Rab GTPase proteins through their interactions with coat components, motors, and SNAREs [6]. Other Ras family members, the Rho GTPases, are also becoming apparent players in membrane transport events by recruiting proteins regulating actin-driven processes, for example, the nucleation promoting factors of the Wiskott-Aldrich Syndrome protein (WASp) family and in turn the ARP2/3 complex, specifically in the late secretory pathway and during endocytic events [1,7,8,9,10].

The family of Rho GTPase proteins is composed of 21 members in mammalian cells and are major regulators of the cytoskeleton [11]. Many Rho GTPases, including RhoA, RhoB, and Cdc42, cycle between an active GTP-bound form and an inactive GDP-bound form. However, ″atypical″ members, RhoU, RhoV, RhoF, RhoD, RhoH; as well as the Rnd subfamily comprising Rnd1/RhoS, Rnd2/RhoN, and Rnd3/RhoE; and the RhoBTB proteins, RhoBTB1-3, are regulated by mechanisms other than GTPase cycling. They are constitutively bound to GTP and either possess high intrinsic nucleotide exchange activity or have substitutions in their GTPase domain that prevent GTPase activity. Therefore, their regulation is achieved through gene expression, post-translational modification, and protein interactions [11]. Rho GTPase activity drives many cellular processes including morphogenesis, cell migration and adhesion, and cell polarity establishment, all of which rely on a tight cooperation between cytoskeletal dynamics and membrane trafficking events [12,13,14,15]. Indeed, one of the earliest family members to be linked with membrane traffic processes was RhoD [16,17,18]. Two members of the RhoBTB family, namely RhoBTB1 and RhoBTB3, have also been implicated in membrane traffic [19,20]. In terms of specific roles for the Rho family in regulating membrane traffic events between the ER and Golgi, Cdc42 is the best characterised. It has an established role in regulating retrograde transport of carriers coated with coat protein complex I (COPI) back to the ER in the early secretory pathway via its observed binding to coatomer [21,22]. Early in transport carrier formation, active Cdc42 at the *cis*-Golgi inhibits the interaction of coatomer and dynein, allowing cargo proteins to be concentrated in one area, and stimulating actin assembly for carrier formation. As coatomer binds cargo receptor proteins, such as p23, and transport carrier formation is completed, Cdc42 dissociates from coatomer, halting actin polymerisation and allowing dynein recruitment and motility [21,22]. Via its COPI interaction, Cdc42 has been shown to be an important modulator of COPI anterograde trafficking through the Golgi by sorting anterograde cargo and influencing membrane curvature to promote COPI tubule formation [23].

This prompted us to ask whether other members of the Rho GTPase family may also play a role in assisting trafficking events throughout the endomembrane system, and so we applied an RNA interference (RNAi) screening approach, targeting each Rho GTPase family member in turn, combined with confocal microscopy and quantitative image analysis, to answer this question.

## 2. Materials and Methods

### 2.1. Antibodies and siRNA Molecules

Antibodies were purchased as follows: anti-mouse Sec31A antibody (BD Biosciences, San Jose, CA, USA), anti-mouse GM130 antibody (BD Biosciences), anti-mouse and anti-rabbit EEA1 antibody (BD Biosciences and Cell Signaling Technology respectively), anti-mouse LAMP1 antibody (DSHB, Iowa City, IA, USA), anti-mouse α-COP antibody (Santa-Cruz Biotechnology, Dallas, TX, USA), anti-mouse myc antibody (Cell Signaling Technology, Danvers, MA, USA), and anti-rabbit Rab5 antibody (Cell Signaling Technology). The following antibodies were used for immunoblotting: anti-rabbit Cdc42 antibody (Santa-Cruz Biotechnology), anti-rabbit RhoBTB3 antibody (Proteintech, Manchester, UK), mouse anti-GAPDH antibody (Abcam, Cambridge, UK), anti-rabbit alkaline phosphatase antibody (Sigma, St. Louis, MO, USA), and anti-mouse alkaline phosphatase antibody (Sigma). RNA oligomers used in depletion experiments were purchased from Ambion (Thermo Fisher Scientific, Waltham, MA, USA) and are listed in Table S1. Two siRNA oligomers targeting the same transcript were pooled together.

### 2.2. DNA Plasmids

An mCherry-RhoBTB3 expression plasmid was made by sub-cloning RhoBTB3 open reading frame from the pEGFP-C1 vector (from Francisco Rivero, University of Hull, Hull, UK) into the pmCherry-C1 (Takara Bio, Kusatsu City, Shiga Prefecture, Japan) using the *Eco*RI and *BgI*II restriction sites. The myc-RhoBTB1 plasmid was a gift from Francisco Rivero, University of Hull, Hull, UK.

### 2.3. Cell Culture and Transfections

HeLa Kyoto cells (human cervical cancer cell line, RRID:CVCL_1922) and U-2 OS cells (human osteosarcoma cell line, ATCC HTB-96) were cultured in Dulbecco’s modified Eagle medium (DMEM) (Life Technologies, Carlsbad, CA, USA) supplemented with 10% foetal calf serum (FCS) (Life Technologies) and 1% L-Glutamine (Life Technologies) and incubated at 37 °C in a humidified atmosphere of 5% CO_2_/95% air. HeLa cells stably expressing YFP-tagged p24 were cultured as above in the presence of 0.5 µg/mL puromycin (Life Technologies). Upon reaching 80% confluency, cells were passaged at a 1:10 dilution. Cells were used from passage 1 to 10, after which they were discarded. Oligofectamine transfection reagent (Life Technologies) was used for the transfection of cells for 48–72 h with Silencer Select small interfering RNAs (siRNAs), following the manufacturer’s protocol. For the screening experiments and follow up studies, two siRNAs targeting the same mRNA at different sites were used. DNA constructs were transfected into cells using Transit-LT1 transfection reagent (LT-1) (Mirus Bio, Madison, WI, USA) following the manufacturer’s protocol.

### 2.4. Immunofluorescence

Cells were fixed with 3% paraformaldehyde (PFA) (Sigma) in phosphate-buffered saline (PBS) warmed to 37 °C for 20 min at room temperature, at which point PFA was removed and cells were quenched with 30 mM glycine (Fisher Scientific, Hampton, NH, USA) in PBS for 5 min. Cells were permeabilised for 5 min in 0.1% Triton X-100 (Sigma) in PBS, and immunostaining was subsequently carried out. The nucleus was stained using 0.2 µg/mL Hoechst 33342 (Sigma) diluted in PBS and coverslips were mounted in Mowiol (Sigma) on glass slides.

### 2.5. Confocal Image Acquisition and Analysis

Confocal images with a resolution of 1024 × 1024 pixels were acquired on a FluoView FV1000 confocal laser scanning microscope (Olympus, Tokyo, Japan), equipped with a 60× UPLSAPO 1.35 NA oil immersion objective (Olympus), in sequential scanning mode, and with a 2× or 3× zoom depending on the experiment carried out. The pixel dwell time was 12.5 μs and images were processed with three times Kalman line averaging. All images were saved in Olympus Original Imaging Format (OIF), which includes greyscale TIF file data. Unbiased, automatic quantifications of images for analysis of organelle number and intensity were performed with CellProfiler 2.1.1 software [24] pipelines, in which individual cells were segmented to perform per cell measurements.

### 2.6. Polar Distribution Score Analysis

Images of cells immunostained for GM130 were analysed with CellProfiler [24]. Briefly, cells were segmented and membrane structures of interest in each cell were detected. The Cartesian coordinates of the fragments were converted into polar coordinates, and the angles were normalised to a zero mean angle. Each cell was divided into eight equal sections and the histogram of frequencies of Golgi fragments was calculated. The frequencies in each bin were subtracted from the theoretical equal distribution frequency of 0.125 (1/8). The sum of absolute differences was presented as the polar distribution score. The minimum value of 0 represents equal frequencies of fragments in each bin, and the maximum value of 1.75 represents all fragments concentrated in a single bin. The analysis script is available online at http://github.com/tilenkranjc/polards. The mean polar distribution score was calculated for each siRNA treatment and normalised to the negative control. Statistical analyses were made using the R statistical package (R Development Core Team 2008).

### 2.7. Live Cell Imaging Experiments and Analysis

Prior to imaging, DMEM was replaced with phenol red-free imaging medium (containing pH-indicator free DMEM (Life Technologies) supplemented with 1% FCS) and returned to the 37 °C incubator for 10 min to allow temperature equilibration. Next, the cells were transferred to an Andor Revolution spinning disk confocal microscope (Oxford Instruments, Belfast, UK) at 37 °C with a humidified atmosphere with 5% CO_2_/95% air and equipped with a 100× UPLSAPO 1.40 NA oil immersion objective (Olympus). Time-lapse videos (512 × 512 pixels) were acquired using Andor-IQ software (IQ version 2.2.1). The resulting videos were processed using ImageJ software. A − 10 subtraction was applied across each video, followed by a Gaussian blur filter with a value of 1 and pixel values were multiplied by 3. The videos were saved for further analysis with Imaris software (Bitplane, Zürich, Switzerland) to extract information on speed and displacement length of p24-YFP carriers in cells across the various treatments. Statistical analysis was carried out on tracks with a displacement length mean greater than 2 µm. Statistical analysis of 3 independent experiments shown as mean ± s.e.m. was carried out and included a Mann Whitney U test, with a Bonferroni post-hoc test, performed against the control non-silencing (NEG) siRNA. For the analysis of p24-YFP carrier morphology, a carrier was considered tubular when its length was equal or greater than 2 µm; when the length of a carrier was less than 2 µm, it was considered vesicular [25].

### 2.8. Total RNA Extraction, cDNA Synthesis, and Real-Time Quantitative PCR

HeLa Kyoto cells were seeded in 12-well plates and transfected with 10 pmol of siRNA (Ambion) for 48 h using Oligofectamine (Life Technologies) according to the manufacturer′s protocol. Total RNA from cells was purified using the Invisorb Spin Cell RNA mini kit (Invitek Molecular, Berlin, Germany). RNA concentrations were determined using a NanoDrop3000 (Thermo Scientific, Waltham, MA, USA). cDNA synthesis was performed with 500 ng of total RNA using the High Capacity cDNA Reverse Transcription kit (Life Technologies) according to the manufacturer’s protocol. Real-time qPCR was performed using Fast SYBR green PCR MasterMix (Life Technologies) in a 7500 FAST real-time PCR system (Life Technologies). One-twentieth of the cDNA reaction was used as a template for the reaction and 200 nM of each primer was used. Three biological replicates were performed. The results were obtained using the −ΔCt method, with mRNA levels from specific siRNA-treated cells being normalised to those found in cells treated with non-silencing (NEG) siRNAs.

### 2.9. Shiga-Like Toxin-1 B Subunit and Transferrin Uptake Assays

HeLa Kyoto cells were seeded on glass coverslips and siRNA transfected as described above. Recombinant wild-type Shiga-like toxin B-chain (SLTxB) was purified as described previously [26] and was labelled with Cy3 according to the manufacturer’s protocol (Amersham, GE Healthcare, Chicago, IL, USA). Following a 72 h siRNA depletion, cells were incubated on ice with 1.5 μg/mL SLTxB in DMEM without 10% FCS for 30 min, and then washed twice with cold PBS to remove unbound toxin followed by incubation of 0, 30, and 240 min at 37 °C with DMEM supplemented with 10% FCS. Cells were fixed in 3% PFA and nuclei stained. Images were acquired on a Leica DMI6000B inverted wide-field microscope equipped with a 63 × 1.25 NA oil immersion objective. A minimum of 100 cells were analysed per siRNA treatment, and the proportion of cells showing SLTxB in the nuclear envelope was recorded as described previously [26]. An analysis of variance (ANOVA) test, with a Bonferroni post hoc test, was performed comparing the data between the negative control and the target gene. The results are presented as mean ± s.e.m. from three independent experiments.

Following a 72 h siRNA depletion, cells were washed three times with ice cold live cell imaging solution (LCIS) containing 20 mM glucose and 1% bovine serum albumin (BSA). Cells were then incubated on ice in LCIS containing 25 µg/mL Alexa Fluor-568 transferrin (Thermo Fisher Scientific) for 30 min. Time point 0 cells were washed in acid wash and PBS and fixed in PFA for 20 min. The remaining coverslips were washed in ice cold PBS to remove the unbound transferrin and incubated at 37 °C in complete medium for the various time points. Before fixation, cells were washed with ice cold acid wash and PBS and fixed in 3% PFA. Immunostaining was carried out as described above and coverslips were mounted on glass slides. Cells were imaged on an Olympus FV1000 confocal microscope equipped with a 60 × 1.35 NA oil immersion objective. Three experimental replicates were carried out.

### 2.10. Protein Extraction and Western Blotting

Cells were lysed using lysis buffer diluted in ultrapure water containing 1% TNS (70 mM Tris (pH 7.4), 150 mM NaCl (Sigma), 0.05% SDS, 1% Triton X-100) and supplemented with 1× Complete™ protease inhibitor cocktail solution (Roche, Basel, Switzerland). The lysates were agitated on a rotary wheel for 30 min at 4 °C. Lysates were centrifuged at 14,000× rpm (20,800× *g*) for 30 min at 4 °C and the soluble fraction was transferred to a new low-protein binding tube. Samples were stored at −20 °C until further use. The protein concentration was quantified using the bicinchoninic acid (BCA) protein assay kit (Thermo Fisher Scientific). Then, 20 μg of protein lysates was boiled at 95 °C with loading buffer (100 mM Tris-Cl (pH 6.8) with 4% sodium dodecyl sulphate, 0.2% bromophenol blue and 20% glycerol), and 200 mM dithiothreitol (DTT) reducing reagent for 5 min. Samples were separated by SDS-polyacrylamide gel electrophoresis (PAGE) on a 12% acrylamide gel. Separated proteins were then transferred onto a nitrocellulose membrane using a wet transfer system (Hoefer, Holliston, MA, USA) set to run for 3 h at 100 mA. Following the transfer, the membrane was Ponceau (Sigma)-stained for 5 min. The membrane was blocked in wash buffer with 5% milk for 1 h at room temperature and probed with the primary antibody overnight. The next day, the membrane was washed in wash buffer and probed with the appropriate alkaline phosphatase (AP)-tagged secondary antibody for 1 h at room temperature. Following this, membranes were incubated with Attophos reagent (Promega, Madison, WI, USA), and imaged on a LAS 4000 (Fujifilm, Tokyo, Japan) gel documentation system. Bands from immunoprecipitation blots were quantified using ImageJ, and data were analysed by a Student′s *t*-test.

## 3. Results

### 3.1. Depletion of Several Rho GTPase Proteins Affects Golgi Complex Morphology

**Figure 1:**
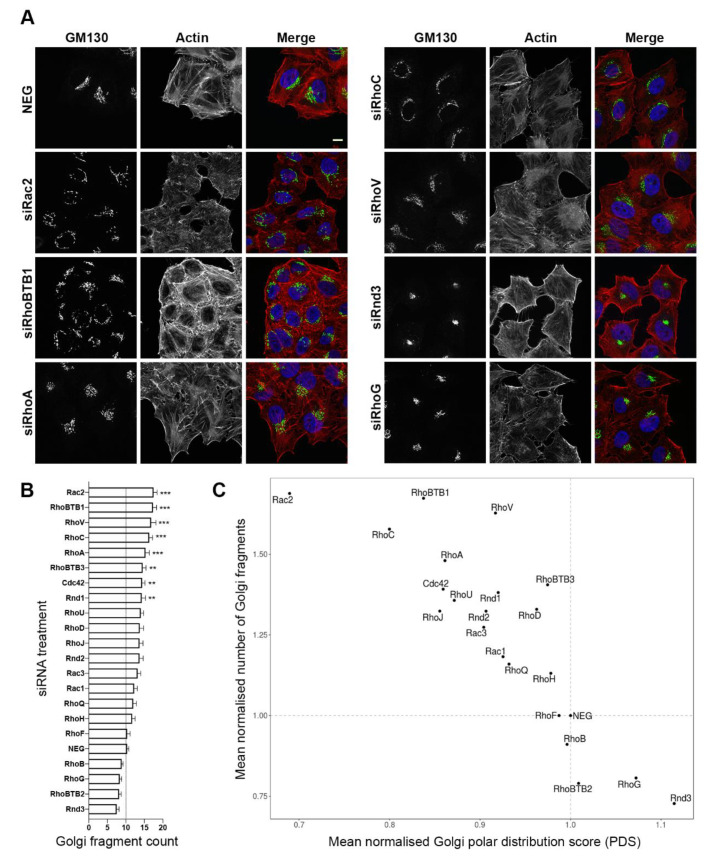
Depletion of several Rho GTPase proteins affects Golgi complex morphology. (**A**) Representative images of HeLa Kyoto cells treated with either non-silencing (NEG) control siRNAs, or siRNA pools targeting specific Rho family GTPases. The *cis*-Golgi is shown in green (marked by GM130), the nuclei are shown in blue, and actin is in red (marked by phalloidin). Bar represents 10 μm. (**B**) Graph showing numbers of detected Golgi fragments in cells following siRNA treatment as indicated. *** *p* < 0.001 and ** *p* < 0.01 compared with NEG control cells. (**C**) Scatter plot showing the mean normalised Golgi complex polar distribution score on the *x*-axis and the mean normalised number of Golgi fragments on the *y*-axis following Rho GTPase depletion for 48 h. *n* = 3 independent experiments, with a total of at least 70 cells analysed per siRNA treatment.

To identify potential Rho GTPases playing a role in the regulation of the endomembrane system, we first focused on the Golgi complex, given that it is a central organelle in the cell, and has transport pathways that connect it to most other membrane compartments. In HeLa Kyoto cells, we systematically depleted the 21 Rho GTPase proteins using a pool of two small interfering RNAs (siRNAs) against each target gene for 48 h (Table S1). We then analysed the morphological status of the Golgi complex by immunostaining for the matrix protein GM130, a marker of the *cis*-Golgi (Figure 1A). Two metrics were initially chosen to quantitatively describe the Golgi complex morphology—firstly, a count of the number of distinct Golgi fragments, and secondly, a novel metric that we termed the Golgi polar distribution score (PDS). Measurement of the Golgi PDS would allow quantification of the distribution of the Golgi and its fragments, after siRNA treatment, allowing us to assess whether the Golgi remained in its typical juxtanuclear position, or became more scattered across the cell. Using this methodology, after normalising the data to cells exposed to non-silencing control siRNAs (NEG), cells with dispersed Golgi fragments would have a PDS of between 0 and 1.0, whereas cells with increased juxtanuclear or compacted Golgi elements would have a score greater than 1.0 (Figure S1). Initial analysis of the images from these experiments revealed that a number of the siRNA treatments resulted in a severe Golgi fragmentation phenotype (Figure 1A). In particular, depletion of Rac2, RhoBTB1, RhoV, RhoC, and RhoA was observed to have the strongest effect (*p* < 0.001) (Figure 1B). In the case of the Rac2 depletion, phalloidin staining of the actin cytoskeleton also revealed a general reduction in the abundance of actin stress fibres in the cells, whereas depletion of RhoBTB1 resulted in an apparent increase in cortical actin. Depletion of RhoBTB3, and to a lesser extent, Cdc42, also caused a small increase in Golgi fragmentation compared with control NEG siRNA-treated cells. Conversely, Rnd3, RhoBTB2, and RhoG depletion each resulted in a decreased Golgi fragmentation (Figure 1B). Analysis of the area occupied by the Golgi, or Golgi fragments, after each siRNA treatment further confirmed the apparent Golgi compaction phenotype, with depletion of Rnd3 and RhoG causing the greatest decrease in area occupied within the cell (Figure S2). The mean number of Golgi complex fragments and Golgi PDS was calculated for each RNAi experiment and normalised to the control (NEG siRNA-treated cells). This analysis revealed a negative correlation between the number of Golgi fragments and Golgi polar distribution score (Figure 1C). The increase in Golgi complex fragmentation was thus seen to be accompanied by fragment dispersion around the cell, whereas a decrease in the number of Golgi complex fragments could be visualised in the form of Golgi compaction.

Both RhoBTB1 and RhoBTB3 have previously been shown to affect Golgi morphology upon depletion [19,20], reinforcing the validity of our quantitative RNAi screening approach. However, with all RNAi-based screens, it is important to confirm the efficacy of each siRNA reagent. The screen was performed using two pooled siRNA sequences targeting each gene product and repeated three times. Real-time quantitative PCR (RT-qPCR) was thus used to confirm the efficiency of the depletions using these pooled siRNAs (Figure S3A,B). In most cases, a knockdown efficiency of at least 80% was achieved, with siRNA pools against RhoH, RhoD, RhoB, and RhoBTB2 causing at least a 60% decrease in the target mRNA. RhoJ RNA expression was not detected in control HeLa Kyoto cells, and thus was assumed not to be expressed, and so was not considered further. Each of the pooled siRNAs that resulted in a statistically significant fragmentation of the Golgi (Figure 1B) was then tested individually. This experiment revealed that all 16 of these reagents (targeting 8 genes) were effective in reducing mRNA levels of their corresponding target by at least 70% (Figure S3C). Overall, these experiments supported our findings on Golgi phenotypes, confirming that our RNAi reagents were effective in depleting gene activity of their respective targets.

### 3.2. Cdc42 and RhoBTB3 Depletion Causes Changes to p24-YFP Carriers in the Early Secretory Pathway

**Figure 2:**
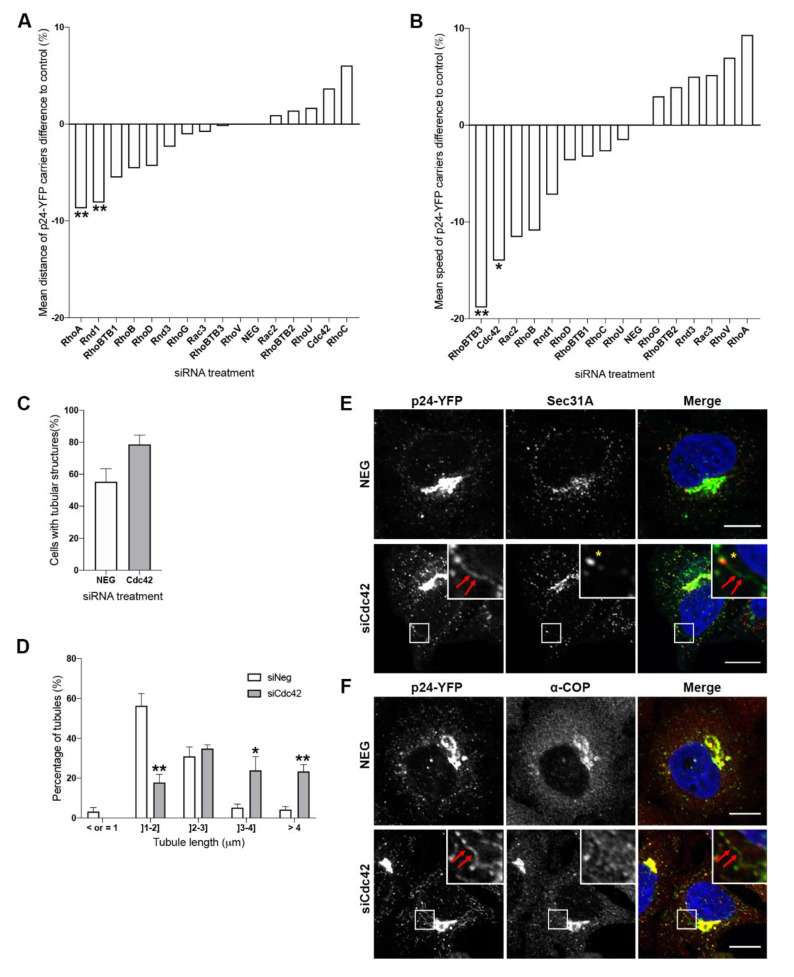
RhoBTB3 and Cdc42 depletion affects the transport of p24-YFP carriers. (**A**) Graph showing the difference in mean distance travelled by p24-YFP carriers in cells depleted of Rho GTPase proteins, as a % of the distances travelled in control cells (NEG siRNA-treated cells). (**B**) Graph showing the difference in mean speed of p24-YFP carriers in cells depleted of Rho GTPase proteins, as a % of the speed travelled in control cells (NEG siRNA-treated cells). ** *p* < 0.01 and * *p* < 0.05 compared with NEG control cells. *n* = 3 independent experiments with at least 17 cells analysed per treatment. (**C**) Graph showing the percentage of cells containing tubular carriers in control and Cdc42-depleted cells. *n* = 3 independent experiments, with at least 28 cells analysed in total. (**D**) Graph showing the percentage of tubular carriers of indicated sizes in NEG siRNA-treated cells (white bars) and Cdc42-depleted cells (grey bars). ** *p* < 0.01 compared with NEG control cells. *n* = 3 independent experiments with at least 64 cells analysed in total. (**E**) HeLa cells stably expressing p24-YFP and treated with either NEG siRNAs or siRNAs targeting Cdc42 were stained with antibodies against Sec31A. Red arrows indicate a tubular carrier extending from an endoplasmic reticulum (ER) exit site (ERES) marked by anti-Sec31A (yellow asterisk). (**F**) Cells stably expressing p24-YFP and treated with either NEG siRNAs or siRNAs targeting Cdc42 were stained with antibodies against α-COP. Red arrows indicate a tubular carrier that is devoid of the COPI coat. Bars represent 10 μm.

As changes to Golgi morphology may be indicative of alterations in membrane transport to or from this compartment, the efficiency of transport between the ER and the Golgi complex was assessed using a HeLa cell line stably expressing the p24 transmembrane cargo receptor protein fused to YFP [25]. The p24 family of molecules are transmembrane proteins found on COPI- and COPII-coated carriers [27,28]. They have long been proposed to function as cargo receptors, in quality control, and in transport along the secretory pathway. They have also been shown to be important for Golgi structure [29,30]. In live cells, they are seen actively shuttling between the ER and the Golgi complex in the form of both vesicular and tubular membrane structures [25]. The Rho GTPases identified as having significant fragmentation effects on Golgi morphology, namely, Cdc42, Rac2, RhoBTB1, RhoBTB3, RhoA, RhoC, RhoV, and Rnd1, as well as other members of the family, RhoU and RhoD, which have been found to play a role in membrane traffic in other studies [31,32], were depleted for 48 h from HeLa cells stably expressing p24-YFP. Following siRNA treatment, the live cells were imaged continuously on a spinning disk confocal microscope for 1 min and the resulting videos were analysed to extract information on mean speed and displacement distance of the p24-YFP carriers (Figure 2). Compared with control cells, the depletion of two proteins, RhoA and Rnd1, was found to significantly decrease the distance travelled by the carriers (Figure 2A). By contrast, RhoBTB3 and Cdc42 depletion resulted in the largest decrease in carrier speed compared with control cells (Figure 2B, and live cell videos S1, S2, S3). Effective depletion of these two proteins (to less than 15% of the levels found in control cells) was confirmed by western blotting analysis (Figure S4). Given that the results from the Golgi morphology screen (Figure 1) and the live cell analysis experiments (Figure 2) both implicated a role for Cdc42, RhoBTB1, and RhoBTB3 in Golgi-associated pathways or function, combined with the fact that all are expressed at high levels in HeLa cells (Figure S3B), we decided to focus our attention on these three GTPases for the remainder of the study.

Upon Rho GTPase depletion, phenotypic differences were noted between the motile carriers in the control (NEG) siRNA-treated cells and Cdc42-depleted cells. In control cells, most of the motile carriers were punctate in shape, whereas in Cdc42-depleted cells, many motile carriers appeared tubular in shape (Figure 2, and live cell video S2). Population analysis revealed that 55% of control cells displayed p24-YFP tubular carriers. In contrast, Cdc42 depletion resulted in a strong increase in the frequency of tubular carriers, with 79% of cells displaying p24-YFP tubular carriers (Figure 2C). Further analysis revealed that over 50% of the p24-YFP tubular carriers in Cdc42-depleted cells were larger than 3 µm in length compared with less than 10% in control cells (Figure 2D), while the majority of p24-YFP carriers in control cells were 1–2 µm in length. Visual inspection of the live cell videos revealed that several of the p24-YFP tubular structures extended from the Golgi, however, tubular structures extending from punctate structures in the peripheral cytoplasm were also visible (Figure 2E, yellow asterisk and red arrows). Co-staining for the ER exit site (ERES) marker Sec31A revealed that these tubular structures extended from ERES in the periphery of Cdc42-depleted cells (Figure 2E). Furthermore, co-staining of these cells for subunits of the COPI coat, specifically α-COP, revealed that the tubular carriers were devoid of the COPI coat (Figure 2F), similar to what has been reported previously for these carriers [25]. Interestingly, active Cdc42 has been shown to promote tubular carrier formation in the Golgi complex during anterograde transport [23] and prevent carrier displacement in the retrograde pathway by competing with dynein for binding to the COPI subunit ƴ-COP, allowing the carrier to form [21,22,33]. Here, we show that Cdc42 is important for carrier formation at the Golgi complex, but also at ERES, and that loss of Cdc42 results in an increase in tubular carrier formation between these two organelles.

### 3.3. RhoBTB3 Localises to Transport Carriers in the Early Secretory Pathway

**Figure 3:**
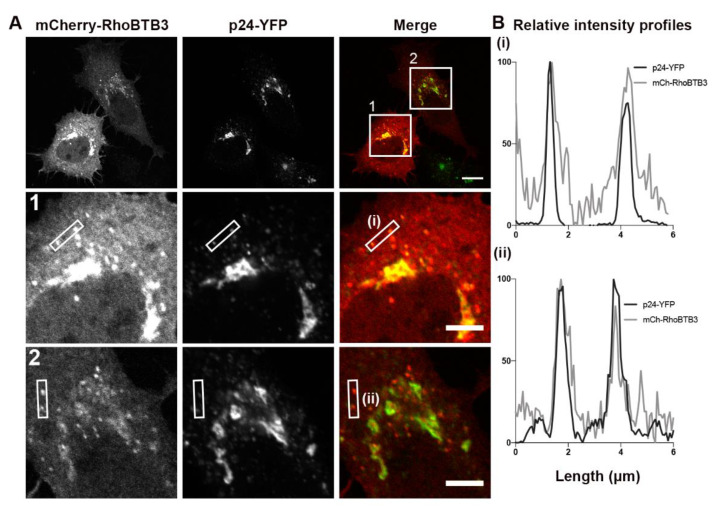
RhoBTB3 localises to transport carriers in the early secretory pathway. (**A**) HeLa cells stably expressing p24-YFP were transiently transfected with mCherry-RhoBTB3 for 18 h and imaged live. In the merge image, mCherry-RhoBTB3 is in red and p24-YFP in green. Bar represents 10 μm. Squares 1 and 2 show zoomed images of two transfected cells. Bars represent 5 μm. (**B**) Fluorescence intensity profiles of the areas marked by rectangles in A1 and A2, showing the correlation of mCherry-RhoBTB3 and p24-YFP signals.

In addition to Cdc42, RhoBTB3 depletion was also found to significantly decrease p24-YFP carrier speed in the early secretory pathway compared with control cells (Figure 2B and live cell video 3). RhoBTB3 has been shown to localise and act at the *cis*-Golgi complex, where it controls the G2/S phase of cell cycle progression by regulating the degradation of cyclin E via the scaffolding protein Cullin 3 at the *cis*-Golgi [19]. Furthermore, it is also reported to interact with the small GTPase Rab9 during endosomal transport to the *trans*-Golgi network (TGN), where it was proposed to be important for carrier docking at the TGN [34]. Overexpression of mCherry-RhoBTB3 in cells stably expressing p24-YFP followed by confocal microscopy revealed the presence of mCherry-RhoBTB3 in juxtanuclear-localised membranes. In addition, co-localisation of distinct punctate mCherry-RhoBTB3 structures with p24-YFP carriers was also observed (Figure 3A). Intensity profile plots were carried out in cells expressing various levels of mCherry-RhoBTB3, and strong overlap of signals of mCherry-RhoBTB3 and p24-YFP was seen on these punctate structures (Figure 3B). Together, these data suggest that not only do the levels of RhoBTB3 influence transport events in the early secretory pathway, but also that a proportion of this protein is physically localised to ER-Golgi carriers.

### 3.4. RhoBTB1 Is Important for Retrograde Transport of the B Subunit of Shiga-Like Toxin-1

The RhoBTB subfamily is composed of three members RhoBTB1, RhoBTB2, and RhoBTB3. In our initial RNAi screen (Figure 1), the depletion of both RhoBTB1 and RhoBTB3 was seen to affect Golgi morphology. Interestingly, however, unlike RhoBTB3, the depletion of RhoBTB1 was not found to significantly affect transport events (as judged by p24-YFP motility) in the early secretory pathway. We thus hypothesised that the Golgi fragmentation observed may result from disrupted membrane flow in the retrograde direction between the endosomes and the Golgi complex.

**Figure 4:**
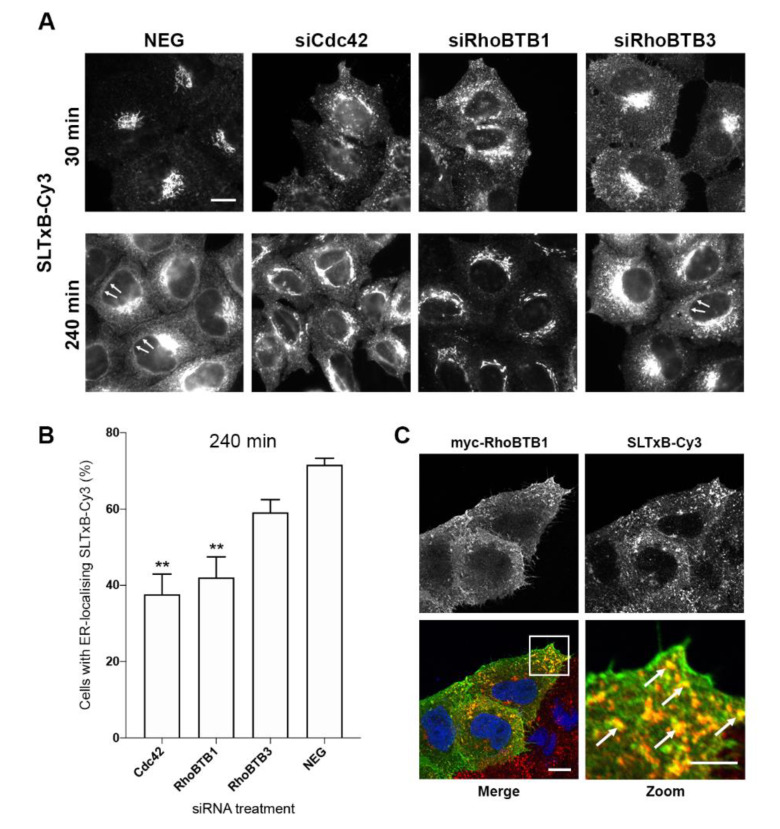
RhoBTB1 depletion affects retrograde transport of SLTxB-Cy3. (**A**) Representative images of HeLa Kyoto cells treated with siRNAs for 72 has indicated and incubated with SLTxB-Cy3 for 30 or 240 min. In NEG control siRNA-treated cells, SLTxB-Cy3 is present at the Golgi complex after 30 min of incubation and at the ER after 240 min of incubation at 37 °C, as seen by the presence of the nuclear envelope signal (arrows). Bar represents 10 μm. (**B**) Graph of the mean % of cells displaying ER-localising SLTxB-Cy3 after 240 min of incubation in cells treated with siRNAs for 72 h, as indicated. Results are presented as mean ± s.e.m. of three independent experiments, with a total of at least 220 cells analysed. ** *p* < 0.01 compared with NEG control cells. (**C**) HeLa Kyoto cells were transfected with a DNA construct encoding myc-RhoBTB1 for 18 h and then incubated with SLTxB-Cy3 for 30 min. Arrows indicate myc-RhoBTB1 (green) and SLTxB-Cy3 (red) co-localising punctate structures. Bars represent 10 μm (merge) and 5 μm (zoom).

The non-toxic cell binding B subunit of *Escherichia coli* Shiga-like toxin-1 (SLTxB) labelled with Cy3 is a well-established tool for monitoring of the retrograde transport pathway from the cell surface to the ER, via endosomes and the Golgi complex [26]. RhoBTB1, RhoBTB3, and Cdc42 were individually depleted from HeLa Kyoto cells and a time course monitoring SLTxB-Cy3 transport from the plasma membrane to the ER was carried out. Cdc42 has previously been reported as being important for SLTxB retrograde transport from the plasma membrane to the ER, and so it was considered as a positive control [35,36]. After 30 min of SLTxB-Cy3 internalisation, control NEG siRNA-treated cells displayed a distinct Golgi localising SLTxB-Cy3 signal, and by 240 min, a clearly visible nuclear envelope pattern could be seen, indicative that the toxin subunit had traversed the retrograde pathway and arrived at the ER (Figure 4A, arrows). As expected, in Cdc42-depleted cells, at 30 min, SLTxB-Cy3 was found predominantly in punctate structures with very few cells displaying a Golgi-like pattern. At 240 min after internalisation in Cdc42-depleted cells, SLTxB-Cy3 showed a strong Golgi-like signal and punctate structures were found throughout the cytoplasm. In these cells, no clear ER pattern was detected, and comparatively few cells showed the toxin in the nuclear envelope, suggestive of a delay in retrograde transport to the ER. In RhoBTB3-depleted cells, similar to that seen in NEG control cells, a strong Golgi-like SLTxB-Cy3 signal was seen at 30 min, and by 240 min, cells displayed an ER and nuclear envelope pattern of SLTxB-Cy3 (Figure 4A, arrows). By contrast, in RhoBTB1-depleted cells, at 30 min, a proportion of the SLTxB-Cy3 was found in a juxtanuclear Golgi-like pattern, however strikingly, large amounts of the toxin seemed to be present in small punctate structures, many of which were in the periphery of the cell (Figure 4A). After 240 min of internalisation, the SLTxB-Cy3 showed a strong Golgi-like signal, however, no ER pattern was visible, indicating a reduction in transport of the toxin between the plasma membrane and the ER (Figure 4A).

To quantify these phenotypes, cells in the population displaying toxin in the nuclear envelope (indicative of transport to the ER) after 240 min were counted. This analysis revealed that, in control NEG siRNA-treated cells, 71% of cells could be seen to have an SLTxB-Cy3-positive nuclear envelope signal (Figure 4B). Depletion of RhoBTB3 resulted in a slight reduction in delivery of toxin to the ER, but this was not statistically significant. However, depletion of Cdc42 and RhoBTB1 resulted in only 40% of the cells displaying a SLTxB-Cy3 nuclear envelope signal (Figure 4B), consistent with the visual observation that, in these cells, the toxin showed an altered distribution. To understand this phenotype a little further, the toxin uptake experiments were repeated, but in cells expressing a myc-tagged version of RhoBTB1. When these cells were fixed, 30 min after toxin internalisation, again, the toxin was found to be mainly localised to peripheral punctate structures, many of which were decorated with myc-RhoBTB1 (Figure 4C, arrows). Together, these results suggest that RhoBTB1 depletion causes a delay in retrograde transport of SLTxB-Cy3 from the plasma membrane to the ER, and that this effect is likely to be occurring at the level of endosomes.

### 3.5. RhoBTB1 Protein Levels Affect Endosomal and Lysosomal Morphology

**Figure 5:**
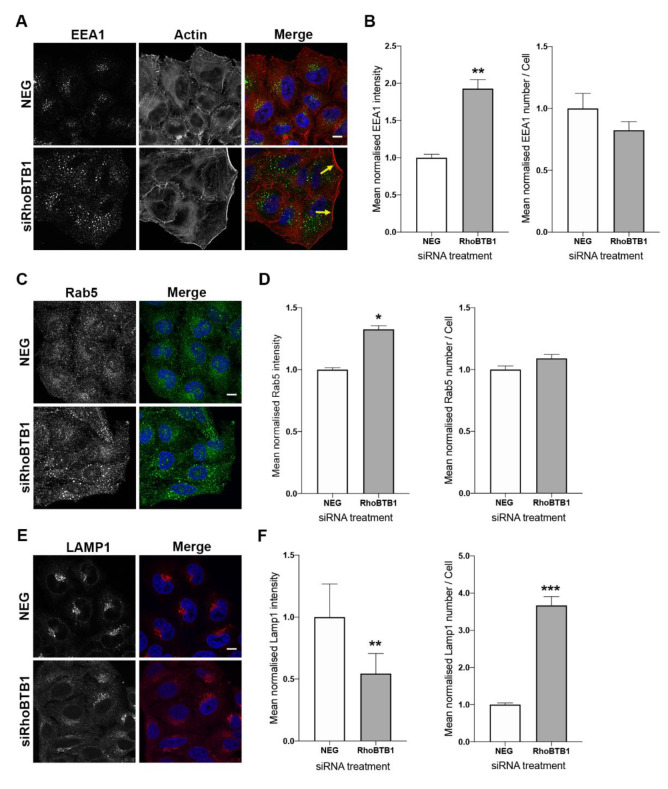
RhoBTB1 is important for architecture of the endosomal-lysosomal system. (**A**) Representative images of the effect of RhoBTB1 depletion on early endosomes and actin cytoskeleton. HeLa Kyoto cells were treated with control (NEG) siRNAs or siRNAs against RhoBTB1 for 72 h. The cells were fixed and immunostained for markers of the early endosomes (EEA1) (green in merged image), stained for the actin cytoskeleton with phalloidin (red) and the nuclei with Hoechst 33342 (blue). Yellow arrows indicate intense staining of actin at the cell periphery. (**B**) Graphs showing the normalised mean intensity of EEA1 and the normalised mean number of EEA1 structures per cell upon siRNA treatment as indicated. Results are presented as mean ± s.e.m. of three independent experiments, with at least 64 cells analysed in total. ** *p* < 0.01 compared with NEG control cells. (**C**) Representative images of the effect of RhoBTB1 depletion on Rab5. HeLa Kyoto cells were treated with control (NEG) siRNAs or siRNAs against RhoBTB1 for 72 h. The cells were fixed and immunostained for Rab5. (**D**) Graphs showing the normalised mean intensity of Rab5 and the normalised mean number of Rab5 structures per cell upon siRNA treatment as indicated. Results are presented as mean ± s.e.m. of three independent experiments, with at least 64 cells analysed in total. * *p* < 0.05 compared with NEG control cells. (**E**) Representative images of the effect of RhoBTB1 depletion on LAMP1. HeLa Kyoto cells were treated with control (NEG) siRNAs or siRNAs against RhoBTB1 for 72 h. The cells were fixed and immunostained for LAMP1. Bars represent 10 μm. (**F**) Graphs showing the normalised mean intensity of LAMP1 and the normalised mean number of LAMP1 structures per cell upon siRNA treatment as indicated. Results are presented as mean ± s.e.m. of three independent experiments, with at least 64 cells analysed in total. *** *p* < 0.001 and ** *p* < 0.01 compared with NEG control cells.

To further study the role of RhoBTB1 in the endocytic pathway, cells were depleted of RhoBTB1 for 72 h, and then fixed and immunostained for early endosomes using anti-EEA1 antibodies. Interestingly, compared with control cells, RhoBTB1-depleted cells displayed brighter EEA1-positive structures, with a dispersed distribution (Figure 5A). Quantitative analysis of these images revealed a significant increase in the intensity of EEA1 per cell, although the actual number of EEA1 structures was similar to those seen in control cells (Figure 5B). Co-staining with phalloidin revealed the presence of a strong cortical actin signal in RhoBTB1-depleted cells compared with control NEG siRNA-treated cells (Figure 5A, yellow arrows). RNAi-mediated depletion experiments using individual, rather than pooled siRNAs targeting RhoBTB1, also showed similar effects on EEA1 (Figure S5A,B). Furthermore, this striking effect on EEA1-positive endosomes could be recapitulated in an independent cell line, namely in U-2 OS osteosarcoma cells, further supporting this phenotype as being specific (Figure S5C,D). In addition, RhoBTB1 depletion was also found to cause a similar effect on a second marker of early endosomes, the small GTPase Rab5, with cells displaying more intense Rab5-positive structures compared with control cells (Figure 5C,D).

As early endosomes represent an upstream organelle for sorting of certain cargo to degradative compartments, the appearance of lysosomes in cells depleted of RhoBTB1 was observed using antibodies against the lysosomal hydrolase LAMP1. Interestingly, RhoBTB1 depletion had the opposite effect on LAMP1 compared with EEA1, namely causing a decrease in the intensity of LAMP1 structures compared with control cells. Concomitantly, the number of LAMP1 structures detected increased more than 3.5-fold under these depletion conditions (Figure 5E,F). These dramatic changes to early endosomes and lysosomes did not appear to be as a result of RhoBTB1 influencing gene expression, as RT-qPCR experiments in RhoBTB1-depleted cells revealed no significant changes in mRNA levels of EEA1, Rab5, or LAMP1 (Figure S6). Together, this suggests that RhoBTB1 directly modulates endosome function (with consequential effects on lysosomes) directly at the early endosome membrane.

**Figure 6:**
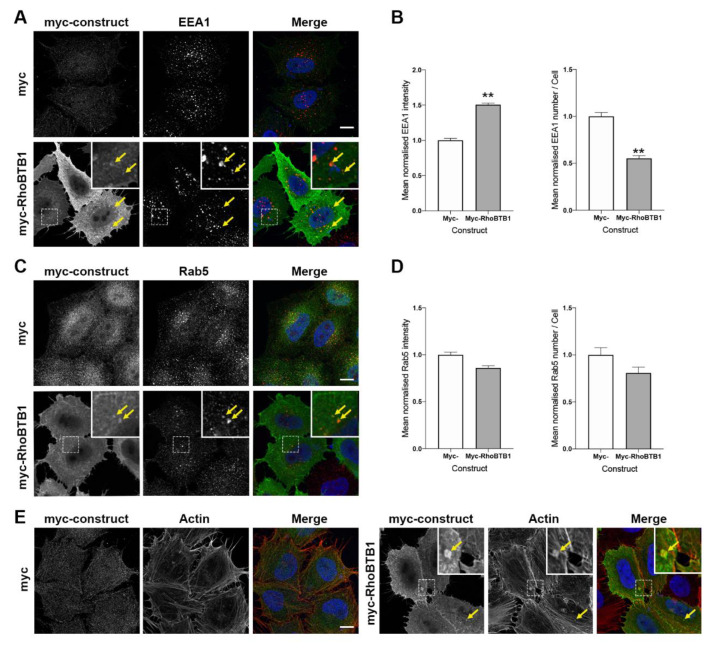
RhoBTB1 localises to early endosomal structures. (**A**) HeLa Kyoto cells overexpressing myc or myc-RhoBTB1 for 18 h were fixed and stained for EEA1. In the merged images, the over-expressed construct is shown in green, EEA1 in red, and the nucleus in blue. Yellow arrows indicate co-localising structures. (**B**) Graphs showing the normalised mean intensity of EEA1 and the normalised mean number of EEA1 structures in overexpressing cells as indicated. Results are presented as mean ± s.e.m. of three independent experiments, with at least 60 cells analysed in total. ** *p* < 0.01 compared with NEG control cells. (**C**) HeLa Kyoto cells overexpressing myc or myc-RhoBTB1 for 18 h were fixed and stained for Rab5. In the merged images, the over-expressed construct is shown in green, Rab5 in red, and the nucleus in blue. Yellow arrows indicate co-localising structures. (**D**) Graphs showing the normalised mean intensity of Rab5 and the normalised mean number of Rab5 structures in overexpressing cells as indicated. Results are presented as mean ± s.e.m. of three independent experiments, with at least 60 cells analysed in total. (**E**) HeLa Kyoto cells overexpressing myc or myc-RhoBTB1 for 18 h were fixed and stained for actin (phalloidin). In the merged images, the over-expressed construct is shown in green, actin in red, and the nucleus in blue. Yellow arrows indicate co-localising structures. Bars represent 10 μm.

As knockdown of RhoBTB1 resulted in distinct morphological changes to endocytic compartments, as well as an altered trafficking pattern of the SLTxB subunit, we next decided to examine the localisation of RhoBTB1 in the endosomal system more carefully, and also to assess any possible effects of it overexpression. Cells were transfected for 18 h with a construct encoding myc-RhoBTB1, after which time they were fixed and immunostained for EEA1 or Rab5. Similar to the scenario in cells depleted for RhoBTB1, the overexpression of myc-RhoBTB1 resulted in a smaller number of more intense EEA1-positive structures compared with that seen in cells expressing myc alone (Figure 6A,B). The overexpression of myc-RhoBTB1 resulted in a slight intensity decrease and reduced number of Rab5-positive structures, but these were not statistically significant (Figure 6C,D). Although myc-RhoBTB1 exhibited a mostly soluble localisation pattern, it was possible to observe its presence on punctate structures containing EEA1 and Rab5 (Figure 6A,C, insets and arrows). Next, the actin cytoskeleton was also visualised by staining cells overexpressing myc-RhoBTB1 with fluorescently-labelled phalloidin. Although the actin was predominantly arranged in fibres spanning the cell, small accumulations of actin co-localising with RhoBTB1 were occasionally seen (Figure 6E, inset and arrows). These observations potentially suggest a direct role for RhoBTB1 in organising early endosome membranes coordinated through the actin cytoskeleton.

### 3.6. RhoBTB1 Depletion Affects Transferrin Uptake and Transport

**Figure 7:**
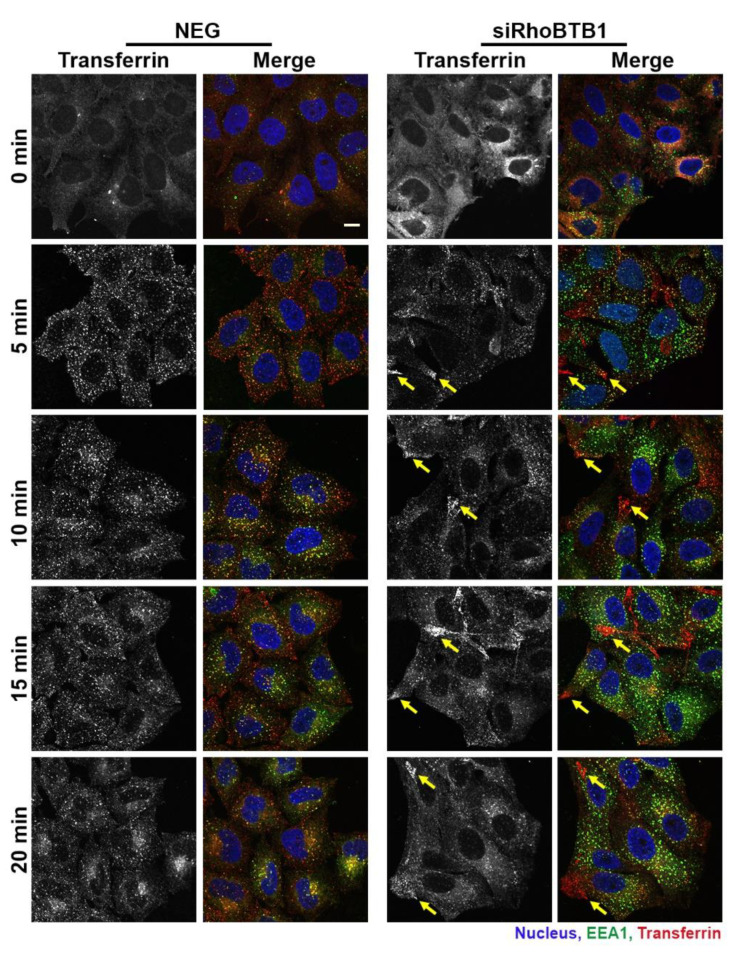
Depletion of RhoBTB1 alters Transferrin-Alexa Fluor 568 trafficking in HeLa Kyoto cells. Representative images of the effect of RhoBTB1 depletion on Tf-Alexa Fluor 568 uptake in HeLa Kyoto cells compared with control NEG siRNA-treated cells. Cells were incubated on ice for 30 min with transferrin (red) before being warmed to 37 °C to allow internalisation to start. Cells were fixed at the times indicated and immunostained for EEA1 (green) and stained for the nuclei (blue). Yellow arrows indicate peripheral accumulations of Tf. Bar represents 10 μm.

Given that changes in RhoBTB1 levels disrupt the morphology of early endosomes, we decided to examine the functional effects of RhoBTB1 depletion on trafficking processes through these organelles. Transferrin (Tf) is a well-established cargo of the recycling endosomal pathway and has been routinely used to study endocytic events. Cells treated with either NEG siRNAs or siRNAs targeting RhoBTB1 were incubated with Tf-Alexa Fluor 568 at 37 °C, and a time course of Tf internalisation was performed (Figure 7). Tf was seen to be internalised rapidly into both control and RhoBTB1-depleted cells, but strikingly, within 5 min, Tf was seen to accumulate in peripheral structures in the depleted cells (Figure 7, arrows), which was a pattern that contrasted with that seen in control cells. Between 10 and 15 min after internalisation, Tf was seen to largely co-localise with EEA1 in control cells, but this was less evident in the depleted cells, where a proportion of Tf remained in peripheral structures. Also noticeable, and in agreement with earlier observations (Figure 5), the EEA1-positive structures were significantly brighter, and by inference, larger in the knockdown cells, compared with control cells. By 20 min, in control cells, Tf had started to accumulate in juxtanuclear structures, most likely the endocytic recycling compartment, whereas in many of the knockdown cells, Tf remained in peripheral structures. Overall, these results suggest that RhoBTB1 levels strongly influence the flux of material through the endocytic pathway, most likely at the level of early endosomes, with consequential effects on downstream recipient compartments.

## 4. Discussion

RNAi screens combined with fluorescent imaging readouts are a powerful means to identify genes important for organelle morphology and function. In the work presented here, we implemented a strategy to identify Rho GTPase family members associated with endomembrane system function by initially focussing on morphological changes to the Golgi apparatus in response to the systematic depletion of each family member in turn. Our initial results found that Cdc42 levels were critical for normal Golgi architecture, as well as membrane traffic through this organelle. This Rho family member has been previously reported to play a critical role at the Golgi [23], and so we were reassured that our approach was a reliable strategy that could assess all Rho GTPases in an unbiased way. In addition to Cdc42, we also discovered that reduced levels of RhoBTB3 modulated Golgi architecture. On initial review of this result, we were not surprised by this finding, given that this molecule has previously been implicated in transport steps between the endosomes and the Golgi through an interaction with Rab9 [34,37]. However, more recent work has implicated a role for RhoBTB3 at the ER in processes associated with inhibiting proteasomal degradation of serotonin receptors [38]. In addition, in a megakaryocyte RhoBTB3-knockout cell line, secretion of alpha-granules was also seen to be altered [39], although this particular work did not identify the subcellular site of the defect. Other RNAi screening work from our own laboratory did not identify RhoBTB3 as a regulator of constitutive secretory transport [31], and so it seems that its role in the early secretory pathway is subtle. In our study here, not only did we find that depleted levels of RhoBTB3 reduced the speed of carriers at the ER-Golgi interface, but also that a proportion of these carriers seemed to contain RhoBTB3. These findings clearly implicate RhoBTB3 in transport events beyond those previously noted in the endosomal system, but further investigation will be needed to identify its interaction network at the ER-Golgi interface, which in turn should shed light on its specific function in this part of the cell.

Our screen revealed that, on depletion, aside from Rac2, RhoBTB1 caused the most severe Golgi fragmentation and dispersal phenotype, an observation also reported previously [20]. Although RhoBTB1 levels seem to have profound effects on the organisation of the Golgi complex, other studies have shown that, in fact, the primary subcellular localisation of this protein (using a GFP-tagged variant) is to punctate and ruffle-like structures in the cell periphery [40], although the exact nature of these has not been determined. Given that RhoBTB1 depletion did not affect the kinetics of transport carriers at the ER-Golgi interface, we turned our attention to a possible role for this protein between the plasma membrane and Golgi complex. Transport studies, in RhoBTB1-depleted cells, revealed that loss of RhoBTB1 had a profound effect on the transport efficiency of SLTxB to the Golgi, and ultimately the ER. In addition, many RhoBTB1 punctate structures were found to co-localise with SLTxB at early time points of internalisation, suggestive of a localisation in the early endocytic pathway. Furthermore, depletion of RhoBTB1 caused striking morphological changes to early endosomes, marked with either the early endosomal tethering factor EEA1 or Rab5, specifically causing an increased intensity and dispersion of these markers. By contrast, downstream organelles, in particular lysosomes marked with LAMP1, exhibited the opposite effect under these conditions. These observations are suggestive of a role for RhoBTB1 in either early endosome fusion events or trafficking of material from early endosomes to downstream compartments. Depletion of RhoBTB1 seemed to be inhibitory to the transfer of transferrin to recycling endosomes, as well as inhibitory to the transport of SLTxB towards the Golgi apparatus. As the initial uptake of both of these ligands seemed to be relatively normal, this points to a direct role for RhoBTB1 at early endosomes, a hypothesis supported by the fact that a number of the myc-RhoBTB1 structures co-localised with both EEA1 and Rab5. These RhoBTB1 structures are likely to be the same as those observed by others [40].

Why should the depletion of RhoBTB1 have such an impact on both the architecture of the Golgi complex, and the apparent size and distribution of lysosomes? Early endosomes act as intermediate organelles between the TGN (and thus Golgi complex) and lysosomes, through bidirectional vesicle exchange, which allows transport of hydrolases and their receptors between these compartments. In turn, these molecules are ultimately responsible for regulating the pH and functionality of the endosomal-lysosomal system [41]. This suggests that RhoBTB1 may play a role in early endosome fusion or sorting, such that its depletion results in aberrant trafficking rates to both lysosomes and the TGN and Golgi complex, with the concomitant effects witnessed. The observed sequestration of actin in the presence of overexpressed RhoBTB1 potentially implicates that this might be the mechanism through which RhoBTB1 works. However, the RhoBTB family of proteins are atypical GTPases, and are not regulated through the conventional pathways of GTP/GDP exchange. Indeed, RhoBTB proteins are known interactors of Cullin 3 (Cul3), an interaction that occurs via the N-terminal region of Cul3 and the first BTB domain within the RhoBTB protein [42]. One hypothesis is that RhoBTB1 is required for the ubiquitin-driven proteasomal degradation of specific early endosomal proteins, in turn allowing the subsequent processing and maturation of endosomal structures. Interestingly, Cul3 has previously been linked to late endosomal maturation, as Cul3 depletion resulted in morphological changes to late endosomes and a defect in the transport of endocytic cargo to lysosomes [43,44,45]. Further work will be needed to see if this is the mechanism and explanation for the results observed in this current study. Regardless, the RNAi and quantitative imaging approach presented here sheds further light on the regulation of transport pathways in cells by Rho family GTPases, and highlights previously unidentified roles for RhoBTB3 at the ER-Golgi interface, and RhoBTB1 in the endocytic pathway.

## Supplementary Materials

The following are available online at https://www.mdpi.com/2073-4409/9/5/1089/s1, Figure S1: Polar distribution score analysis method, Figure S2: Effect of siRNA-mediated depletions on total Golgi area, Figure S3: Validation of the efficiency of siRNA depletions by real-time qPCR, Figure S4: Validation of siRNA-mediated depletion of RhoBTB3 and Cdc42 by western blotting, Figure S5: RhoBTB1 is important for early endosome morphology, Figure S6: RhoBTB1 depletion has no effect on EEA1, LAMP1 and Rab5 mRNA levels, Table S1: siRNA library used, Video S1: Live cell imaging of HeLa cell stably expressing p24-YFP and treated with non-silencing NEG control siRNA, Video S2: Live cell imaging of HeLa cell stably expressing p24-YFP and treated with siRNA targeting Cdc42, Video S3: Live cell imaging of HeLa cell stably expressing p24-YFP and treated with siRNA targeting RhoBTB3.
