# Supplementary material for: RNA Interference Screening Identifies Novel Roles for RhoBTB1 and RhoBTB3 in Membrane Trafficking Events in Mammalian Cells
Source: Cells. 2020 Apr 28;9(5):1089. doi: 10.3390/cells9051089 (PMC7291084; doi:10.3390/cells9051089)
Supplement: Supplementary file 1 [file cells-09-01089-s001.zip › cells-776692-supplementary.pdf]

# RNA Interference Screening Identifies Novel Roles for RhoBTB1 and RhoBTB3 in Membrane Trafficking Events in Mammalian Cells

**Maeve Long [1,2], Tilen Kranjc [1], Margaritha M. Mysior [1] and Jeremy C. Simpson [1,*]**

[1] Cell Screening Laboratory, School of Biology and Environmental Science & Conway Institute for Biomolecular and Biomedical Research, University College Dublin, Belfield, Dublin 4, Ireland; maeve.long@ucdconnect.ie (M.L.); tilen@tilenkranjc.com (T.K.); margaritha.m.mysior@ucdconnect.ie (M.M.M.); jeremy.simpson@ucd.ie (J.C.S.)

[2] Current address: Translational Stem Cell Biology and Metabolism Program, Faculty of Medicine, Biomedicum Helsinki, University of Helsinki, Haartmaninkatu 8, Helsinki 00290, Finland

* Correspondence: jeremy.simpson@ucd.ie; Tel.: +353-1-716-2345

## Supplementary Data

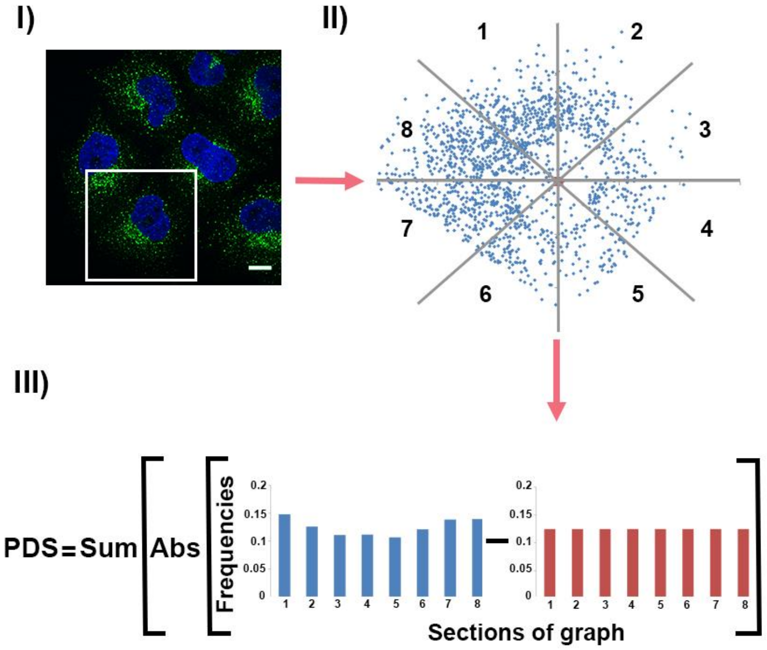


**Supplementary Figure 1:** Polar distribution score analysis method. (**I**) Cluster of HeLa cells immunostained for a membrane marker of interest (green) and the nuclei stained with Hoechst 33342 (blue). The cell in the white insert is used as an example. Bar represents 10 μm. (**II**) The coordinates of each membrane structure in the cell of interest shown in (**I**) are recorded using the cell centre as coordinate (0; 0) and their position is represented on a graph divided into 8 equal sections. The cell is rotated such that section 1 corresponds to the section with the most structures. (**III**) The number of structures in each section is counted and a histogram (shown in blue) representing the relative frequency of each structure is generated. Each histogram generated is compared to the histogram of a synthetic cell with "equal structure polar distribution" (shown in red) to produce the polar distribution score (PDS).

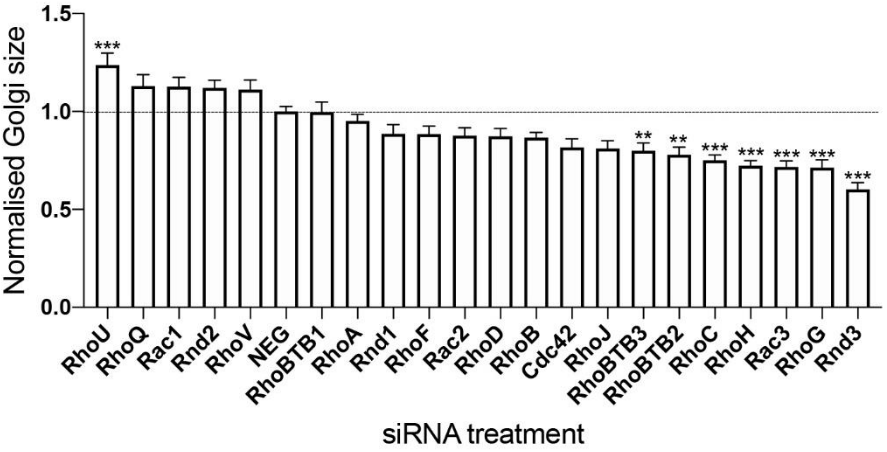


**Supplementary Figure 2:** Effect of siRNA-mediated depletions on total Golgi area. Graph showing total area occupied by detected Golgi fragments (marked by GM130), normalised to that in cells treated with NEG non-silencing control siRNAs, following siRNA treatment for 48 hours. n=3 independent experiments, with a total of at least 70 cells analysed per siRNA treatment. ***$p<0.001$ and **$p<0.01$ compared to NEG control cells.

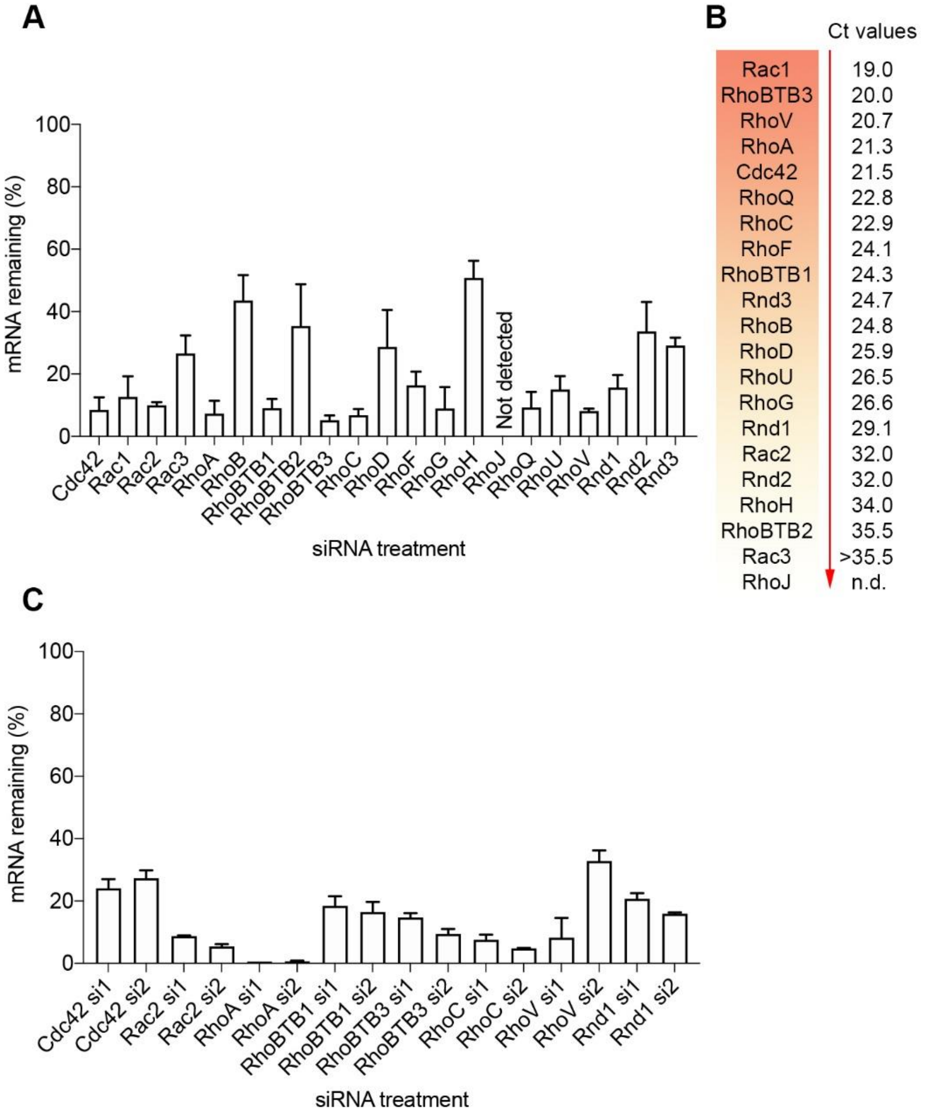


**Supplementary Figure 3:** Validation of the efficiency of siRNA depletions by real-time qPCR. (**A**) Validation of the efficiency of the siRNA depletions using pooled siRNAs judged at the mRNA level by real-time qPCR. Bars indicate relative mRNA levels remaining in cells after 48h of siRNA transfection compared to control NEG siRNA-treated cells. Results are presented as mean ± s.e.m. of 3 independent experiments each containing 4 replicates. (**B**) Graphic showing the respective Ct values at which each mRNA was detected in control cells (in decreasing abundance) by real-time qPCR. Transcripts of RhoJ were not detected (n.d.). (**C**) Validation of the efficiency of the siRNA depletions using individual siRNAs (si1 and si2) judged at the mRNA level by real-time qPCR. Data only shown for those genes annotated as 'hits' in Figure 1. Bars indicate relative mRNA levels remaining in cells after 48h of siRNA transfection compared to control NEG siRNA-treated cells. Results are presented as mean ± s.e.m. of 3 independent experiments each containing 4 replicates.

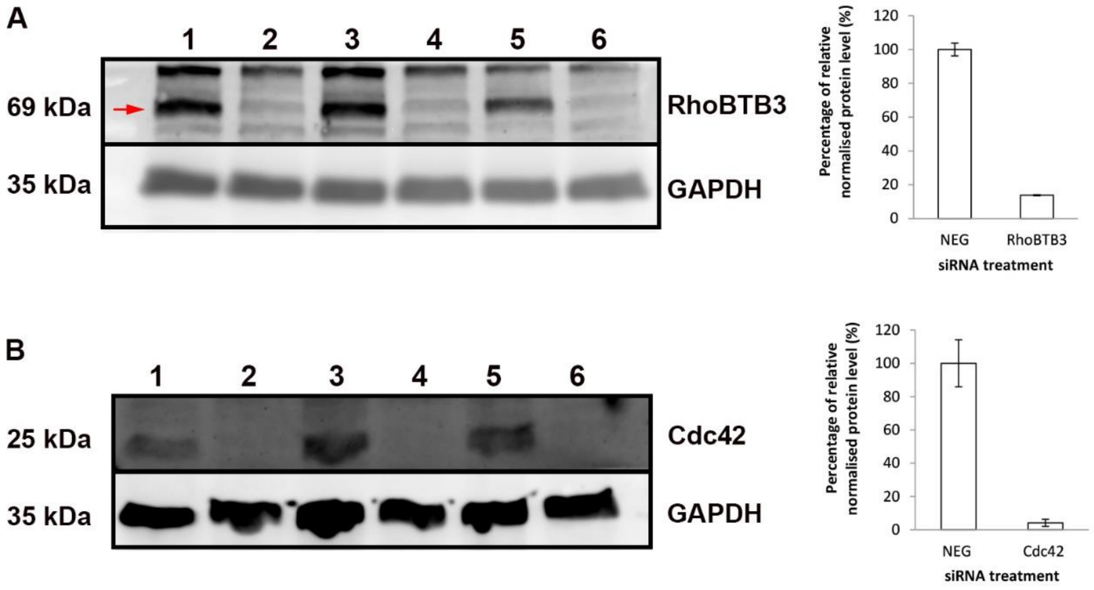


**Supplementary Figure 4:** Validation of siRNA-mediated depletion of RhoBTB3 and Cdc42 by western blotting. Representative images of cell extracts subjected to SDS-PAGE, western blotting and probed for either (**A**) RhoBTB3 or (**B**) Cdc42. GAPDH was used as a loading control and associated densitometry analysis of western blots. Values represent mean and s.e.m. of 3 independent experiments, normalised to GAPDH. Lanes 1, 3 and 5 contain the control NEG siRNA samples, whilst lanes 2, 4 and 6 contain samples treated with siRNAs against RhoBTB3 in (**A**) or Cdc42 in (**B**). Densitometry analysis of protein intensities on the western blots is shown in the graphs on the right.

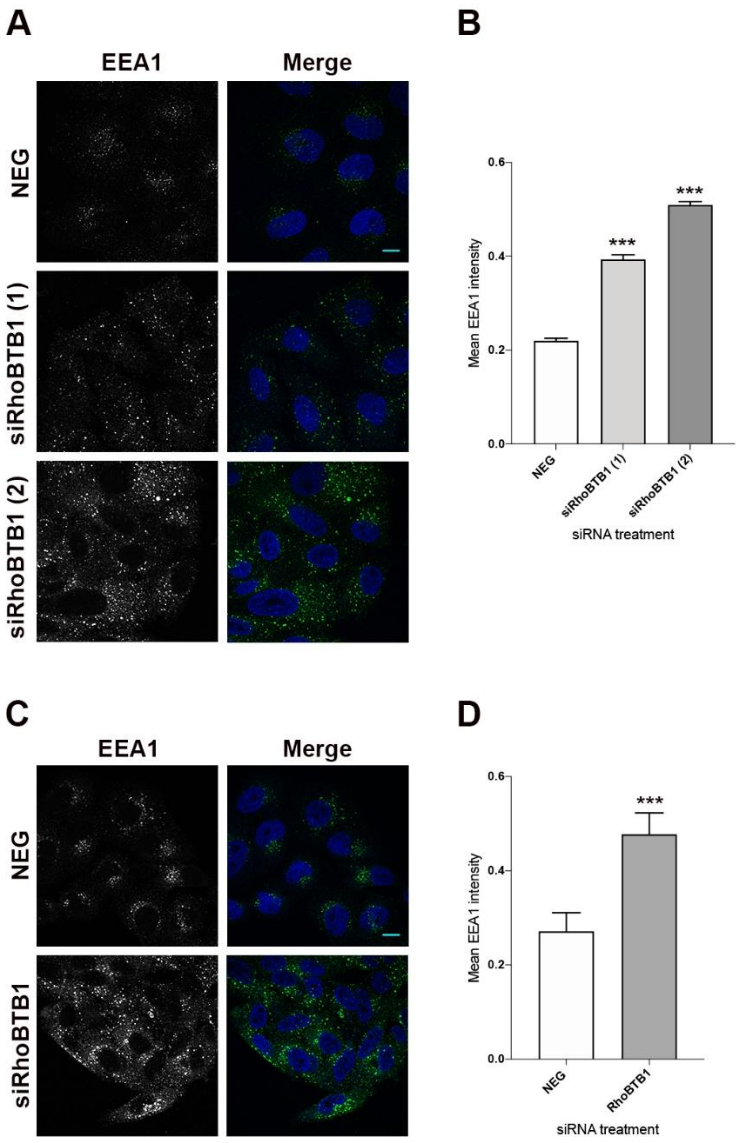


**Supplementary Figure 5:** RhoBTB1 is important for early endosome morphology. (**A**) Representative images of the effect of RhoBTB1 depletion on early endosomes. HeLa Kyoto cells were treated with control (NEG) siRNAs or individual siRNA sequences against RhoBTB1 for 72 hours. The cells were fixed and immunostained for markers of the early endosomes (EEA1) (green in merged image) and the nuclei with Hoechst 33342 (blue). (**B**) Graphs showing the relative mean intensity of EEA1 per cell upon siRNA treatment as indicated. Results are presented as mean ± s.e.m. of 3 independent experiments, with at least 50 cells analysed in total. ***$p<0.001$ compared to NEG control cells. (**C**) Representative images of the effect of RhoBTB1 depletion on early endosomes. U-2 OS cells were treated with control (NEG) siRNAs or siRNAs against RhoBTB1 for 72 hours. The cells were fixed and immunostained for markers of the early endosomes (EEA1) (green in merged image) and the nuclei with Hoechst 33342 (blue). (**D**) Graphs showing the relative mean intensity of EEA1 per cell upon siRNA treatment as indicated. Results are presented as mean ± s.e.m. of 3 independent experiments, with at least 50 cells analysed in total. ***$p<0.001$ compared to NEG control cells. Bars represent 10 μm.

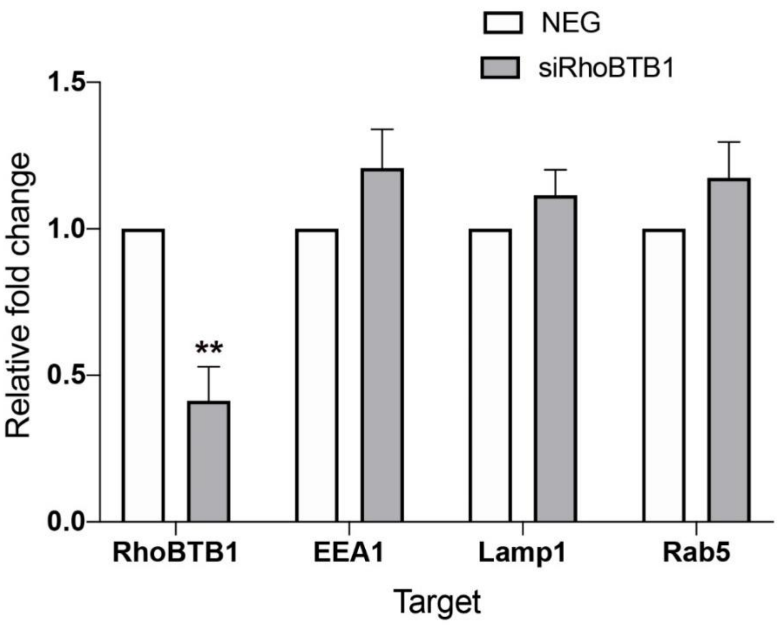


**Supplementary Figure 6:** RhoBTB1 depletion has no effect on EEA1, LAMP1 and Rab5 mRNA levels. Graph showing the effect of 72h of RhoBTB1 depletion on the mRNA levels of EEA1, LAMP1 and Rab5 measured by RT-qPCR and compared to levels found in control NEG siRNA-treated cells. Bars indicate relative mRNA levels. Results are presented as mean ± s.e.m. of 3 independent experiments. **$p$<0.01 compared to NEG control cells.

**Supplementary Table 1:** siRNA library used

Pooled Silencer Select siRNAs used for depletion of the 21 Rho GTPase proteins. All siRNAs were purchased from Ambion, and their unique IDs are detailed below.

| Target gene | siRNA ID (sequence 1) | siRNA ID (sequence 2) |
|---|---|---|
| RhoA, ras homolog gene family, member A | s758 | s759 |
| RhoB, ras homolog gene family, member B | s1574 | s1575 |
| RhoC, ras homolog gene family, member C | s97 | S98 |
| RhoD, ras homolog gene family, member D | s26839 | s26840 |
| RhoF, ras homolog gene family, member F | s29103 | s29104 |
| RhoG, ras homolog gene family, member G | s230023 | s1580 |
| RhoH, ras homolog gene family, member H | s1598 | s1599 |
| RhoQ, ras homolog gene family, member Q | s23824 | s23823 |
| RhoU, ras homolog gene family, member U | s33825 | s33826 |
| RhoV, ras homolog gene family, member V | s46902 | s46903 |
| Rac1, ras-related C3 botulinum toxin substrate 1 | s11711 | s11712 |
| Rac2, ras-related C3 botulinum toxin substrate 2 | s11715 | s11714 |
| Rac3, ras-related C3 botulinum toxin substrate 3 | s11717 | s11718 |
| Cdc42, cell division cycle 42 (GTP binding protein, 25kDa) | s2766 | s2765 |
| Rnd1, Rho family GTPase 1 | s26087 | s26088 |
| Rnd2, Rho family GTPase 2 | s15660 | s15659 |
| Rnd3, Rho family GTPase 3 | s1577 | s1578 |
| RhoBTB1, Rho-related BTB domain containing 1 | s493 | s495 |
| RhoBTB2, Rho-related BTB domain containing 2 | s23322 | s23323 |
| RhoBTB3, Rho-related BTB domain containing 3 | s22463 | s22464 |
| RhoJ, ras homolog gene family, member J | s32981 | s32980 |

**Supplementary Videos**

**Video 1.** Live cell imaging of HeLa cell stably expressing p24-YFP and treated with non-silencing NEG control siRNA. Frame rate is 10 frames/sec.

**Video 2.** Live cell imaging of HeLa cell stably expressing p24-YFP and treated with siRNA targeting Cdc42. Frame rate is 10 frames/sec.

**Video 3.** Live cell imaging of HeLa cell stably expressing p24-YFP and treated with siRNA targeting RhoBTB3. Frame rate is 10 frames/sec.
